# Reduced activity of two LPL C-terminal variants, p.Glu396Val and novel p.Trp417Cys: a clinical, biochemical and structural study

**DOI:** 10.3389/fphys.2026.1820368

**Published:** 2026-06-10

**Authors:** Jun Tang, Qi Huang, Yanchuan Xie, Xueping Qiu, Huawei Wang, Mei Xue, Zhe Dai

**Affiliations:** 1 Department of Endocrinology, Zhongnan Hospital of Wuhan University, Wuhan, China; 2 Central Laboratory, Henan Key Laboratory of Rare Diseases, Endocrinology and Metabolism Center, The First Affiliated Hospital, and College of Clinical Medicine of Henan University of Science and Technology, Luoyang, China; 3 Center for Gene Diagnosis and Department of Laboratory Medicine, Zhongnan Hospital of Wuhan University, Wuhan, China; 4 Department of Clinical Nutrition, Zhongnan Hospital of Wuhan University, Wuhan, China; 5 State Key Laboratory of Metabolism and Regulation in Complex Organisms, College of Life Sciences, Wuhan University, Wuhan, China

**Keywords:** C-mannosylation, C-terminal domain, functional assay, hypertriglyceridemia, lipoprotein lipase, missense variant

## Abstract

**Background:**

Lipoprotein lipase (LPL) is essential for plasma triglyceride (TG) hydrolysis, and biallelic *LPL* loss-of-function variants cause familial chylomicronemia syndrome. However, the clinical and functional significance of heterozygous *LPL* variants remains incompletely characterized. We investigated two heterozygous *LPL* missense variants, c.1187A>T (p.Glu396Val) and the novel c.1251G>C (p.Trp417Cys), in patients with severe hypertriglyceridemia (HTG).

**Methods:**

Two probands underwent comprehensive clinical, biochemical, and imaging assessments. Genetic analysis involved whole-exome sequencing and Sanger confirmation. Variant interpretation incorporated *in silico* prediction, evolutionary conservation, AlphaFold-based residue mapping, LPL-GPIHBP1 structural-context analysis, and NetCGlyc prediction. Functional effects were assessed in HEK293T cells transfected with wild-type or mutant LPL plasmids, measuring mRNA/protein expression and enzymatic activity.

**Results:**

Clinically, Proband 1 exhibited recurrent pancreatitis with moderate hyperglycemia, whereas Proband 2 had extreme HTG without pancreatitis but with severe insulin resistance, reflecting variable expressivity. Two *LPL* missense variants, c.1187A>T (p.Glu396Val) and the novel c.1251G>C (p.Trp417Cys), both located in exon 8. Both affected residues were highly conserved and localized to the C-terminal domain by AlphaFold-based mapping. NetCGlyc predicted Trp417 as a potential C-mannosylation-related residue. Mutant LPL mRNA and protein expression were comparable to wild-type, whereas enzymatic activity was significantly reduced in cell lysates and culture medium.

**Conclusions:**

These findings provide functional evidence that p.Glu396Val and the novel p.Trp417Cys impair LPL enzymatic activity despite preserved protein abundance, supporting a qualitative functional defect. Our findings highlight the value of functional validation and metabolic assessment in interpreting heterozygous *LPL* variants in HTG.

## 1 Introduction

Lipoprotein lipase (LPL) is the rate-limiting enzyme responsible for the hydrolysis of triglycerides (TGs) in circulating chylomicrons and very-low-density lipoproteins (VLDL), thereby playing a central role in plasma lipid homeostasis (Wu et al., 2021; Basu and Goldberg, 2020; Wheless et al., 2024). Impaired LPL function leads to defective TG clearance, resulting in hypertriglyceridemia (HTG), which in severe cases predisposes to recurrent acute pancreatitis and other metabolic complications (Qiu et al., 2023). Persistent triglyceride-rich lipoprotein accumulation and remnant lipoprotein enrichment have been associated with increased atherosclerotic cardiovascular risk, while severe hypertriglyceridemia frequently coexists with hepatic steatosis, obesity, insulin resistance, and type 2 diabetes. These metabolic factors may further aggravate LPL insufficiency and contribute to variable clinical expression among carriers of *LPL* variants (Ginsberg et al., 2021; Baratta et al., 2023).

Genetic defects in *LPL* represent a major cause of monogenic and oligogenic HTG. Biallelic loss-of-function variants result in familial chylomicronemia syndrome, whereas heterozygous variants often display variable expressivity and incomplete penetrance, influenced by environmental and metabolic modifiers such as obesity, insulin resistance, alcohol intake, and pregnancy (Dron and Hegele, 2020; Carrasquilla et al., 2021). With the increasing use of next-generation sequencing, numerous rare *LPL* missense variants have been identified; however, the pathogenic relevance of many such variants remains unclear without functional validation (Carrasquilla et al., 2021; Dron and Hegele, 2020). Heterozygosity for lipoprotein lipase deficiency is associated with an increased risk of hypertriglyceridemia, the severity of which is strongly influenced by secondary metabolic stressors. Nevertheless, triglyceride levels in most affected individuals can be effectively controlled with dietary management, lifestyle interventions, and established lipid-lowering therapies (Hegele, 2025).

Structurally, LPL consists of an N-terminal catalytic domain (residues 1-315) and a C-terminal domain (residues 316-448), which is critical for protein folding, dimerization, binding to heparan sulfate proteoglycans, and interaction with glycosylphosphatidylinositol-anchored high-density lipoprotein-binding protein 1 (GPIHBP1) (Perera et al., 2025). Recent high-resolution structural studies, including the crystal structure of the LPL-GPIHBP1 complex and cryoelectron microscopy structures of dimeric LPL, have highlighted the functional importance of the C-terminal domain in maintaining enzymatic activity *in vivo* (Arora et al., 2019). Notably, several missense variants located in the C-terminal region disproportionately impair LPL secretion or stability rather than directly affecting the catalytic site (Gunn and Neher, 2023). In addition to structural determinants, post-translational modifications play a crucial role in LPL maturation and secretion. C-mannosylation of tryptophan residues has been identified as an important modification for several secreted proteins (Okamoto et al., 2017).

In the present study, we identified two heterozygous *LPL* missense variants, c.1187A>T (p.Glu396Val) and a novel c.1251G>C (p.Trp417Cys), in patients with hypertriglyceridemia. By integrating detailed clinical phenotyping, biochemical assessment, *in vitro* functional assays, and structure-based interpretation, we aimed to elucidate the pathogenic mechanisms underlying these variants.

## 2 Methods

### 2.1 Study subjects and clinical evaluation

Patients were recruited from Zhongnan Hospital of Wuhan University. Clinical data collected included demographic characteristics, body mass index, fasting lipid profiles, oral glucose tolerance test (OGTT) results, and history of acute pancreatitis. Pancreatic CT imaging was reviewed by experienced radiologists. The study was approved by the Institutional Ethics Committee of Zhongnan Hospital of Wuhan University (Ethics Approval No.: 2025368K). Written informed consent was obtained from all study participants.

### 2.2 Genetic analysis

Genomic DNA was extracted from peripheral blood leukocytes. Whole-exome sequencing was performed, and the identified variants were subsequently confirmed by Sanger sequencing. Variant annotation followed the Human Genome Variation Society (HGVS) nomenclature using the reference transcript NM_000237.3. *In silico* pathogenicity predictions were conducted using SIFT (Ng and Henikoff, 2001), PolyPhen-2 (Adzhubei et al., 2013), MutationTaster (Schwarz et al., 2014), and Condel (Gonzalez-Perez and Lopez-Bigas, 2011), while evolutionary conservation was assessed using PhyloP (Pollard et al., 2010) and GERP++ (Davydov et al., 2010).

The candidate variants were interpreted with clinical symptoms and signs according to Standards and Guidelines for the Interpretation of Sequence Variants: A Joint Consensus Recommendation of the American College of Medical Genetics and Genomics and the Association for Molecular Pathology (Richards et al., 2015).

### 2.3 Construction of wild-type and mutant recombination plasmids

pcDNA3.1-H-LPL-WT vector containing normal human *LPL* cDNA was used as template to generate site-directed mutagenesis. The coding sequence regions in *LPL* gene were amplified using primers containing HindIII and Xho I restriction sites. The *LPL* primers were as follow: forward primer 5′-TAGCGTTTAAACTTAAGCTTATGGAGAGCAAAGCCCTGCT-3′ containing Hind III site, reverse primer 5′-ACGGGCCCTCTAGACTCGAGTCAGCCTGACTTCTTATTCA-3′ containing Xho I site. Moreover, the mutation plasmids were generated by PCR-based site-directed mutagenesis. These two mutations (c.1251G>C, c.1187A>T) were introduced into *LPL* cDNA by mutagenic primers. The primers used for site‐directed mutagenesis are listed as follows: pcDNA3.1-H-LPL (G1251C) forward primer 5′-AGTGATTCATACTTTAGCTGCTCAGACTGGTGGAGCAGTC-3′, pcDNA3.1-H-LPL (G1251C) reverse primer 5′-GACTGCTCCACCAGTCTGAGCAGCTAAAGTATGAATCACT-3′; pcDNA3.1-H-LPL (A1187T) forward primer 5′-TCCTTCCTAATTTACACAGTGGTAGATATTGGAGAACTAC-3′, pcDNA3.1-H-LPL (A1187T) reverse primer 5′-GTAGTTCTCCAATATCTACCACTGTGTAAATTAGGAAGGA-3′.

### 2.4 Cell culture and treatments

Human embryonic kidney 293T cells (HEK293T cells), purchased from Wuhan Yousi Biotechnology Co., Ltd., were used for functional analysis of *LPL* variants. HEK293T cells were cultured in Dulbecco’s Modified Eagle Medium (Gibco) containing 10% fetal bovine serum (Gibco) at 37°C and 5% CO2. Wild-type and mutant human LPL recombination plasmids were transfected into HEK293T cells using Lipofectamine 2000 reagent (Invitrogen) according to the manufacturer’s instructions. After culturing for 36 hours, the culture medium was discarded and new medium containing heparin sodium (the final concentration was 20 U/mL) was added. The incubation continued for 1 hour, and then the supernatant and cells were collected, respectively.

### 2.5 RT-PCR

Tansfected HEK293T cells were collected, and the total RNA was extracted using the TRIpure Reagent (ELK Biotechnology). cDNA was synthesized using a EntiLink™ 1st Strand cDNA Synthesis Kit (ELK Biotechnology). Quantitative RT-PCR was performed with a SYBR Green PCR reagent kit (ELK Biotechnology) on a StepOne™ Real-Time PCR system (Life technologies). Finally, the absorption value of SYBR Green fluorescence in each sample was detected. The relative expression level was calculated using 2-ΔΔCt and the mRNA expression of LPL was normalized to β-actin. The primer sequences used were as follows: for LPL 5’-GTCCACCGCAAATGCTTCTA-3’ and 5’-TGCTGTCACCTTCACCGTTC-3’; for β-actin 5’-AAGAACCGCTGCAACAATCTG-3’ and 5’-GGCCTGATTGGTATGGGTTTC-3’.

### 2.6 Western blot

Tansfected HEK293T cells were lysed in a modified radioimmunoprecipitation assay buffer (ASPEN). The concentration of cell lysates was determined using the BCA Protein Assay Kit (ASPEN) after removing cell debris by centrifugation. Protein samples were separated by SDS-PAGE and then transferred to PVDF membranes (Millipore). The membranes were blocked with 5% nonfat milk for 1 hour at room temperature. Primary antibodies (Proteintech) were incubated overnight at 4 °C, and then secondary antibodies (Proteintech) were incubated after washing at room temperature for 2 hours. Protein expression was detected by ECL Chemiluminescence Detection Kit (ASPEN) and an enhanced chemiluminescence detection system (Tanon). ImageJ software was used to quantify the intensities of the protein bands, and the protein expression of LPL was normalized to β-actin.

### 2.7 LPL activity assay

After the transfected HEK293T cells were incubated in a medium containing heparin, the culture medium were harvested and centrifuged for LPL enzyme activity analysis. The remaining precipitated cells were solubilized in cell lysis buffer and the cell lysates were further centrifuged and the supernatant was collected. The samples were then analyzed using the CheKine™ Micro LPL Activity Assay Kit (Abbkine) in accordance with the manufacturer’s instructions.

### 2.8 Structural analysis

Protein secondary structure was predicted using PSIPRED (Jones, 1999). Evolutionary conservation of the affected amino acid residues was assessed by multiple sequence alignment of LPL orthologs using Clustal Omega (Sievers et al., 2011), and sequence conservation was visualized with WebLogo (Crooks et al., 2004). AlphaFold-based residue mapping was performed using the publicly available human LPL model from AlphaFold DB corresponding to UniProt accession P06858 to localize Glu396 and Trp417 within the C-terminal domain (Fleming et al., 2025). Structural visualization was performed using PyMOL (The PyMOL Molecular Graphics System, Schrödinger, LLC).The experimentally determined LPL-GPIHBP1 complex structure (PDB ID: 6E7K) was used to provide interface-level context (Arora et al., 2019). Glu396 was mapped onto the LPL chain, and GPIHBP1 residues within 5 Å and 8 Å were inspected using PyMOL. Potential C-mannosylation-related tryptophan residues were predicted using NetCGlyc (Julenius, 2007).

### 2.9 Statistical analysis

GraphPad Prism 7.0 software was used for statistical analysis. The values are shown as the mean ± standard deviation (SD). One-way analysis of variance (ANOVA) was used for statistical analysis among multiple groups followed by Tukey’s *post hoc* test. *P* < 0.05 was considered statistically significant.

## 3 Results

### 3.1 Clinical characteristics

**Figure 1:**
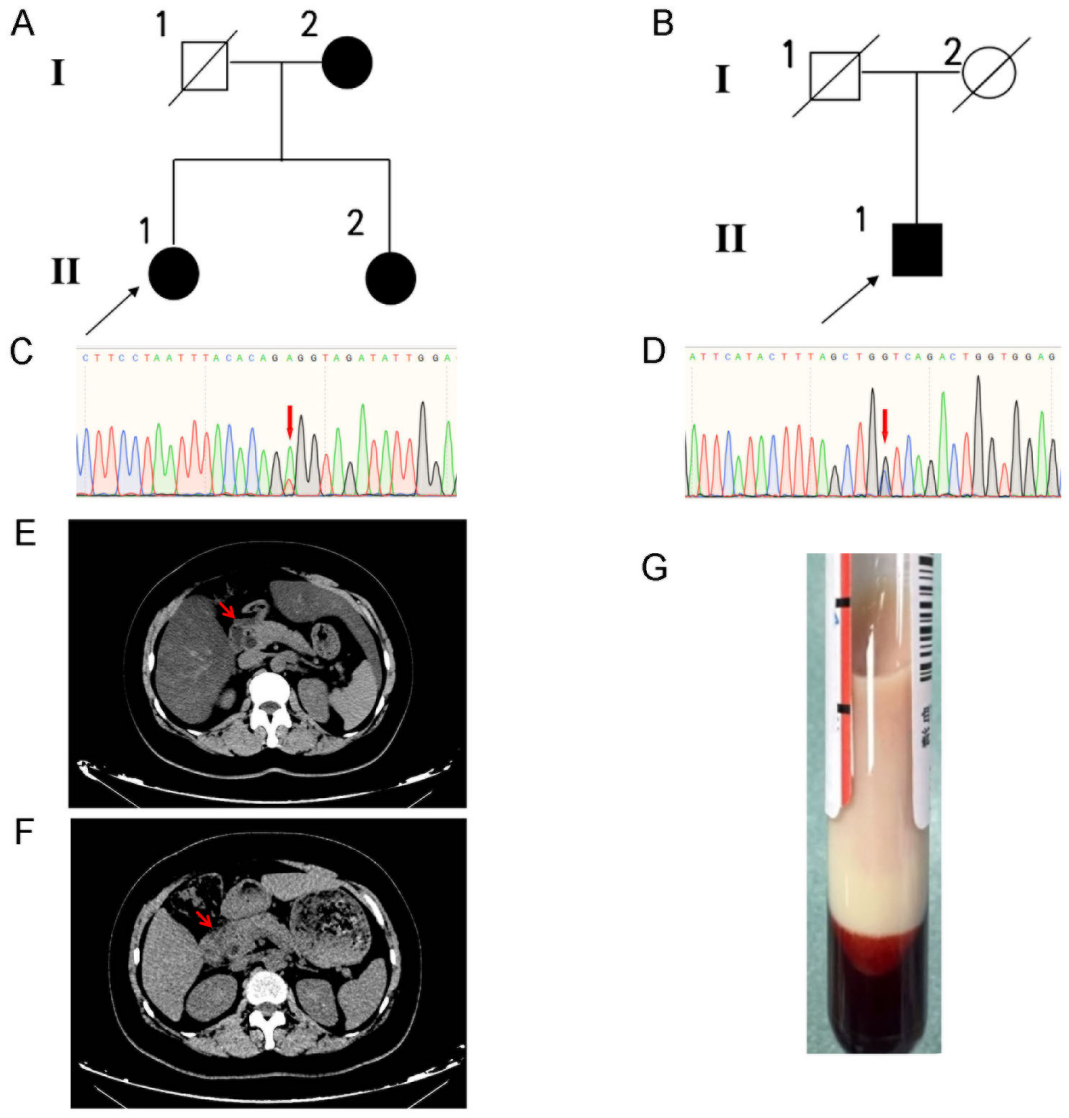
Clinical and genetic features of the two probands carrying heterozygous *LPL* variants. **(A)** Pedigree of the family carrying the LPL p.Glu396Val variant. The proband 1 is indicated by an arrow. Squares represent males and circles represent females. Filled symbols indicate individuals with hypertriglyceridemia and LPL p.Glu396Val variant. **(B)** Pedigree of the family carrying the novel LPL p.Trp417Cys variant. The proband 2 is indicated by an arrow. **(C)** Representative Sanger sequencing chromatograms showing the heterozygous c.1187A>T (p.Glu396Val) variant. **(D)** Representative Sanger sequencing chromatograms showing the heterozygous c.1251G>C (p.Trp417Cys) variant. **(E, F)** Representative abdominal CT images of Proband 1 during episodes of acute pancreatitis. Arrows indicate the swollen pancreas in the upper abdomen, accompanied by peripancreatic inflammatory changes. **(G)** Representative blood sample from Proband 2 showing a grossly lipemic appearance. The serum appears milky and opaque because of marked accumulation of triglyceride-rich lipoproteins, consistent with extreme HTG.

Clinical characteristics of the two probands are summarized in **Table 1**. Proband 1 (**Figure 1A**) is a 48-year-old female (BMI 26.04 kg/m²) with a 5-year history of hyperglycemia and poor glycemic control (HbA1c 10.4%). She has a history of two episodes of acute pancreatitis (hospitalized in 2023 and 2024) (**Figures 1E, F**) and underwent cholecystectomy in 2006. Her family history is notable for dyslipidemia in her mother and sister. Baseline fasting lipid profile demonstrated moderate to severe hypertriglyceridemia (fasting TG 8.24 mmol/L) with low HDL-C (0.70 mmol/L) and relatively low LDL-C (0.89 mmol/L). After lipid-lowering therapy with fenofibrate, TG decreased to 3.79 mmol/L. Her OGTT demonstrated marked hyperglycemia across the curve with preserved C-peptide and insulin responses (**Table 2**), consistent with type 2 diabetes with insulin insufficient control on current therapy.

**Table 1:** Clinical features and lipoprotein profile of the two probands.

Variable	Proband 1	Proband 2
Age (years)	48	51
Gender (male/female)	female	male
BMI (kg/m2)	26.04	28.31
Alcohol intake	no	no
Diabetes mellitus	yes	yes
Hypertension	yes	no
Number of pancreatitis	2	0
Amylase (U/L)	103	42
Lipase (U/L)	265	33
Before treatment		
TG (mmol/L)	8.24	56.90
CHO (mmol/L)	5.84	12.92
HDL-C (mmol/)	0.70	0.16
LDL-C (mmol/)	0.89	0.59
Lipid treatment (after admission)	Fenofibrate	Atorvastatin, Choline Fenofibric Acid Extended-Release Capsules
After treatment		
TG (mmol/L)	3.79	18.28
CHO (mmol/L)	3.94	8.11
HDL-C (mmol/)	0.83	0.52
LDL-C (mmol/)	1.70	1.30

BMI, body mass index; TG, triglycerides; CHO, total cholesterol; HDL-C, high-density lipoprotein cholesterol; LDL-C, low-density lipoprotein cholesterol.

**Table 2:** OGTT, C-peptide and insulin results of the two probands.

		Proband 1			Proband 2	
Time point	Glucose (mmol/L)	C-peptide (ng/mL)	Insulin (mIU/mL)	Glucose (mmol/L)	C-peptide (ng/mL)	Insulin (mIU/mL)
0 min	10.62	2.344	6.604	6.78	3.085	13.11
30 min	12.49	3.102	6.904	13.17	5.077	29.76
60 min	17.04	3.679	15.89	21.38	10.88	63.23
120 min	18.07	4.469	13.67	15.07	8.945	30.29
180 min	16.82	4.243	1.968	9.47	6.350	23.19

OGTT, oral glucose tolerance test.

Proband 2 (**Figure 1B**) is a 51-year-old male (BMI 28.31 kg/m²) who presented for intermittent polyuria/polydipsia; he has a 5-year history of type 2 diabetes and a 7-year history of hyperlipidemia. He has not had pancreatitis. Liver and abdominal ultrasound showed fatty liver and bilateral renal calculi; pancreatic ultrasound revealed no remarkable abnormalities. Baseline fasting triglyceride levels were profoundly elevated (56.90 mmol/L), with markedly lipemic serum was observed at blood sampling (**Figure 1G**), and decreased to 18.28 mmol/L following lipid-lowering therapy (atorvastatin and choline fenofibric acid extended-release). OGTT data show very high post-load glucose peaks with robust C-peptide and insulin responses (**Table 2**), indicating marked insulin resistance with preserved β-cell secretory capacity. He denied a family history of dyslipidemia in his parents.

Taken together, both probands exhibited marked hypertriglyceridemia and dysregulated glucose metabolism; Proband 1 had recurrent acute pancreatitis and poorer glycemic control, whereas Proband 2 had extreme hypertriglyceridemia without pancreatitis but with evidence of severe insulin resistance.

### 3.2 Identification of *LPL* variants

Considering the clinical phenotypes observed in the two probands, a genetic etiology was suspected. Whole-exome sequencing of genomic DNA extracted from peripheral blood leukocytes identified two heterozygous missense variants in the *LPL* gene (**Figures 1C, D**): NM_000237.3:c.1187A>T (p.Glu396Val) in Proband 1 and NM_000237.3:c.1251G>C (p.Trp417Cys) in Proband 2. Both variants were confirmed by Sanger sequencing and were interpreted as presumed germline variants. In Proband 1, the same p.Glu396Val variant was also confirmed in her mother and sister, both of whom had a history of dyslipidemia, supporting familial aggregation among the tested relatives. Nevertheless, because not all relatives were genetically tested and detailed lipid phenotypes were unavailable for every family member, formal segregation analysis remains limited. In Proband 2, the p.Trp417Cys variant was absent from public population databases and affected a highly conserved amino acid residue. As no parental history of dyslipidemia was reported and familial testing was unavailable, the inheritance pattern and possible *de novo* status of this variant remain undetermined. *In silico* pathogenicity prediction consistently classified both variants—NM_000237.3:c.1187A>T (p.Glu396Val) and the novel NM_000237.3:c.1251G>C (p.Trp417Cys)—as deleterious across multiple algorithms, including SIFT, MutationTaster, Condel, and PolyPhen-2. Evolutionary conservation analysis using PhyloP (vertebrates), PhyloP (placental mammals), and GERP++ further indicated that both affected residues are highly conserved (**Table 3**).

**Table 3:** *In silico* pathogenicity predictions and evolutionary conservation analysis of the identified *LPL* variants.

Variant (HGVS)	SIFT	MutationTaster	Condel	PolyPhen-2	PhyloP (Vertebrates)	PhyloP (Placental mammals)	GERP++
c.1187A>T (p.Glu396Val)	Damaging	Disease-causing	Deleterious	Probably damaging	Conserved	Conserved	Conserved
c.1251G>C (p.Trp417Cys)	Damaging	Disease-causing	Deleterious	Probably damaging	Conserved	Conserved	Conserved

### 3.3 Structural interpretation

**Figure 2:**
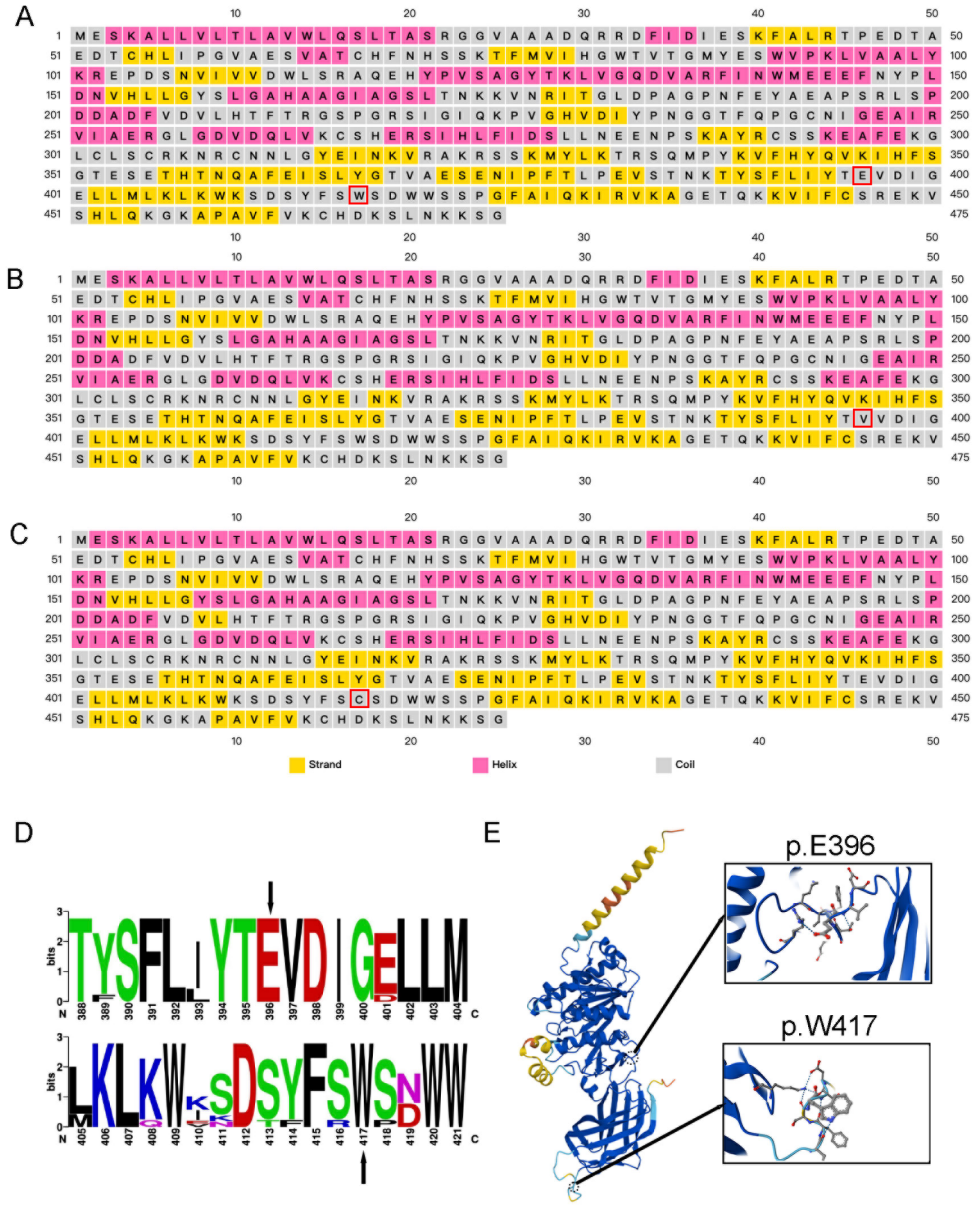
*In silico* structural analysis of wild-type and mutant LPL proteins. **(A–C)** Predicted secondary structures of **(A)** wild-type LPL, **(B)** LPL p.Glu396Val, and **(C)** LPL p.Trp417Cys generated using PSIPRED. The affected residues are indicated by red boxes: Glu396 and Trp417 in WT-LPL, Val396 in p.Glu396Val-LPL, and Cys417 in p.Trp417Cys-LPL. β-strands, α-helices, and random coils are shown in yellow, magenta, and grey, respectively. **(D)** Evolutionary conservation analysis of the LPL protein region encompassing residues 396 and 417 based on multiple sequence alignment of vertebrate orthologs. Conservation scores are visualized using WebLogo, with higher bits indicating stronger conservation. **(E)** AlphaFold-based structural mapping of Glu396 and Trp417 in human LPL. Both residues are localized within the C-terminal domain. Enlarged views show the local structural context of Glu396 and Trp417. Structural visualization and analysis were performed using PyMOL.

Structural mapping localized both p.Glu396Val and p.Trp417Cys to the C-terminal domain of LPL (**Figures 2A–C**). Secondary structure prediction using PSIPRED indicated that the overall α-helix and β-strand organization of the mutant proteins was largely comparable to that of the wild-type LPL. Multiple sequence alignment demonstrated that both Glu396 and Trp417 are highly conserved across vertebrate species (**Figure 2D**). AlphaFold-based structural mapping localized both Glu396 and Trp417 within the C-terminal domain of LPL (**Figure 2E**). Glu396 was positioned within the C-terminal structural region, whereas Trp417 was located within the Trp-rich lipid-binding region. These findings provide structural context for the reduced LPL activity observed *in vitro* but do not directly establish altered folding, dimerization, GPIHBP1 binding, or conformational dynamics. NetCGlyc prediction identified Trp417 as a potential C-mannosylation-related tryptophan residue in WT-LPL, with a prediction score of 0.592. Because NetCGlyc scores represent algorithm-derived prediction values rather than direct probabilities, this result should be interpreted as a positive bioinformatic prediction. Replacement of Trp417 by cysteine removes this tryptophan residue and may therefore abolish this predicted site (**Supplementary Figure 1A**). Mapping onto the experimentally determined LPL-GPIHBP1 complex provided additional interface-level structural context. Glu396 could be visualized in the 6E7K structure, and PyMOL-based interface-proximity analysis showed that it was not located within 5 Å or 8 Å of GPIHBP1 in the static complex (**Supplementary Figure 1B**).

### 3.4 Functional analysis of *LPL* mutants *in vitro*

**Figure 3:**
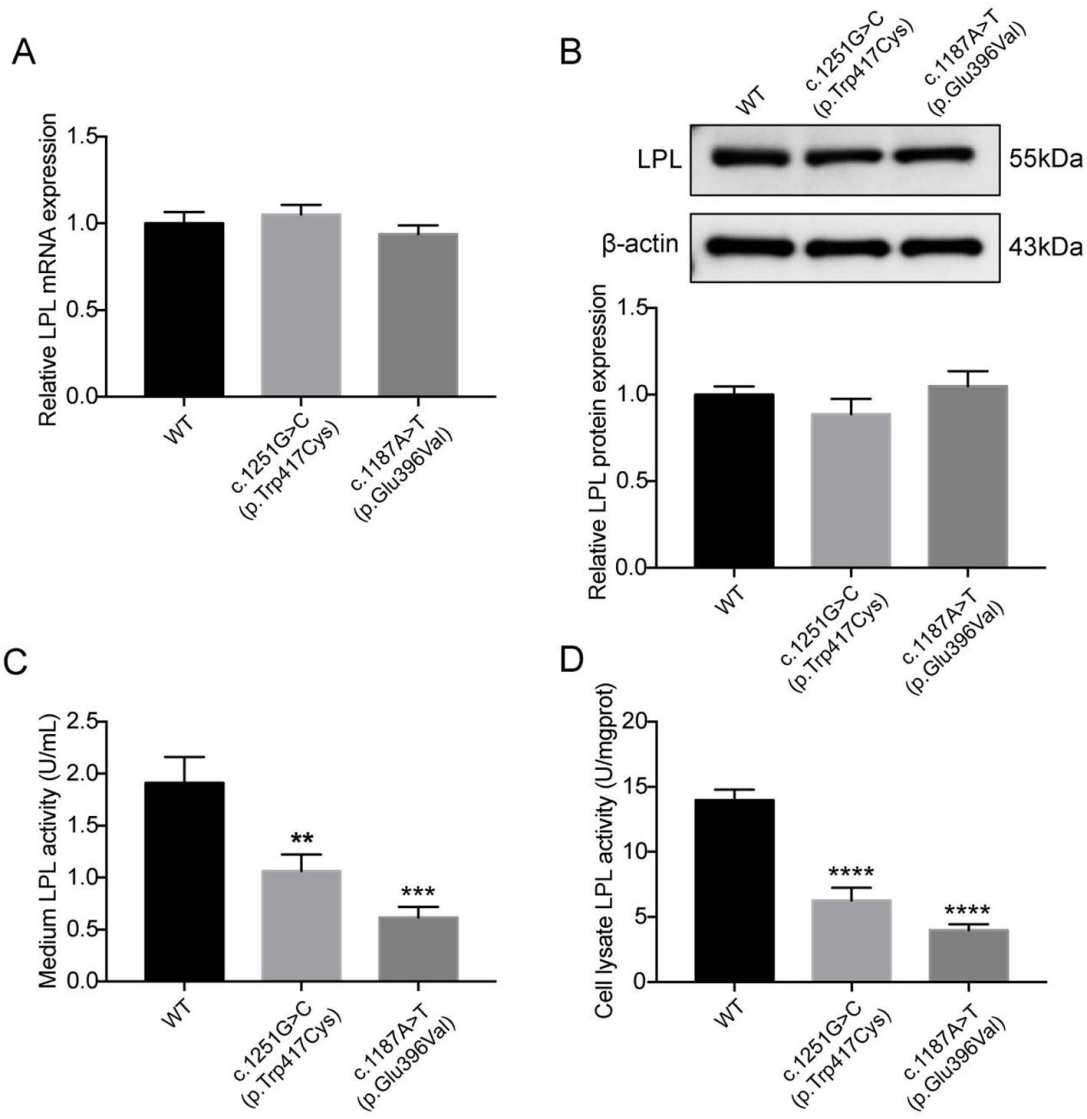
Functional analysis of LPL wild-type and mutant proteins *in vitro*. **(A)** mRNA expression of wild-type and mutant *LPL* genes in HEK293T cells. **(B)** Western blot and quantification of LPL protein expression in HEK293T cells. **(C)** LPL activity of wild‐type and LPL mutants in the cell culture medium. **(D)** LPL activity of wild‐type and *LPL* mutants in the cell lysates. Values are shown as mean ± SD. Compared to WT group,**p < 0.01, ***p < 0.001, ****p < 0.0001.

To evaluate the effect of the *LPL* mutations, HEK293T cells were transfected with plasmids carrying wild-type and mutant *LPL* genes (c.1251G>C and c.1187A>T). As shown in **Figures 3A, B**, no significant differences in the levels of LPL mRNA and protein expression were found among wild-type and mutant plasmid-transfected HEK293T cells, suggesting that neither of these two mutations affects the transcription and translation of *LPL* gene. In addition, we further analyzed LPL enzyme activity in cell culture medium and cell lysate. The results show that HEK293T cells transfected with wild-type LPL plasmids exhibited stable levels of LPL enzyme activity in both cell culture medium and cell lysate. However, the LPL enzyme activity levels in the cell culture medium and cell lysate of HEK293T cells transfected with these two *LPL* mutations (c.1251G >C and c.1187A >T) were significantly lower than those transfected with wild-type LPL (**Figures 3C, D**). Both mutant constructs showed significantly reduced LPL activity compared with WT-LPL under identical experimental conditions. Because empty-vector or untransfected-cell controls were not included in the initial assay, the measured residual activity should be interpreted as relative activity compared with WT-LPL rather than absolute mutant-specific activity.

The *LPL* c.1187A>T (p.Glu396Val) variant fulfills multiple ACMG/AMP criteria for pathogenicity, including PS1 (resulting in the same amino acid change as a previously established pathogenic variant), PS3 (*in vitro* functional studies demonstrating deleterious effects on LPL activity), PM1 (located in a critical functional domain), and PP3 (deleterious predictions by multiple *in silico* algorithms). The *LPL* c.1251G>C (p.Trp417Cys) variant meets multiple ACMG/AMP criteria for pathogenicity: PS3 (functional studies demonstrating impaired LPL activity), PM1 (located in a critical C-terminal lipid-binding domain), PM2 (absent from population databases), PP3 (deleterious predictions by multiple in silico tools), and PP4 (phenotype highly consistent with LPL deficiency).

## 4 Discussion

In this study, we combined clinical phenotyping, biochemical testing, *in vitro* functional assays and structural analyses to evaluate two heterozygous *LPL* missense variants, c.1187A>T (p.Glu396Val) and c.1251G>C (p.Trp417Cys). Our data indicate that both variants impair LPL function and likely contribute to the severe hypertriglyceridemic phenotypes observed in the probands, underscoring the importance of the C-terminal domain in maintaining LPL activity.

Biallelic loss-of-function variants in *LPL* cause familial chylomicronemia syndrome, a rare monogenic disorder with an estimated prevalence of approximately 1 in 1,000,000 (Burnett et al., 1993). In contrast, hypertriglyceridemia is common in the general population, with prevalence estimates ranging from approximately 10% to 30% depending on population characteristics and diagnostic cutoffs (Park et al., 2023). Therefore, the heterozygous *LPL* variants identified in this study should be placed within the broader spectrum of multifactorial hypertriglyceridemia rather than interpreted simply as isolated monogenic causes. Heterozygous *LPL* variants may act as genetic susceptibility factors whose clinical expression is amplified by secondary metabolic stressors, including obesity, insulin resistance, type 2 diabetes, hepatic steatosis, alcohol exposure, or medications. Clinically, LPL dysfunction is most strongly associated with severe hypertriglyceridemia, chylomicronemia, pancreatitis, and increased atherosclerotic cardiovascular risk related to triglyceride-rich lipoproteins and remnant particles. The contrasting presentations of our two probands, recurrent pancreatitis in Proband 1 versus extreme hypertriglyceridemia with marked insulin resistance but no pancreatitis in Proband 2, underscore how partial LPL dysfunction may interact with metabolic context to shape disease severity and clinical outcomes (Dron and Hegele, 2020; Carrasquilla et al., 2021; Ginsberg et al., 2021; Hegele, 2025).

To date, over 250 pathogenic or likely pathogenic *LPL* variants affecting the coding sequence have been reported (Perera et al., 2025). The mature LPL protein comprises 448 amino acids and contains two functional domains: (1) an N-terminal α/β-hydrolase domain harboring a catalytic triad, and (2) a C-terminal lipid-binding domain formed by a β-barrel that mediates interactions with lipids (Kobayashi et al., 2002). The *LPL* gene is located on chromosome 8p21.3 and consists of 10 exons and 9 introns (Perera et al., 2025). The *LPL* gene is located on chromosome 8p21.3 and consists of 10 exons and 9 introns (Perera et al., 2025). Both variants identified in this study are in exon 8 within the C-terminal domain of LPL. This exon encodes a functionally relevant region that includes the Trp-rich lipid-binding segment (residues 412-422), where Trp417 is located (Perera et al., 2025). We summarized representative C-terminal *LPL* variants with reported clinical phenotypes and functional evidence in **Supplementary Table 1**. These include pathogenic or likely pathogenic variants associated with familial chylomicronemia syndrome, severe hypertriglyceridemia, pancreatitis, impaired secretion, reduced enzymatic activity, or altered GPIHBP1-related interaction, as well as the two variants identified in the present study. Importantly, benign variants were also included, indicating that the C-terminal domain is not uniformly intolerant to amino acid substitution. Instead, pathogenicity in this region appears to be residue- and context-dependent.

Unlike previously reported *LPL* variants that reduce LPL abundance by impairing mRNA or protein expression, p.Glu396Val and p.Trp417Cys showed preserved LPL mRNA and protein levels but significantly reduced enzymatic activity, indicating a predominantly qualitative rather than quantitative functional defect. A similar genotype-function dissociation has been reported for selected *LPL* variants, such as Leu252Val, which impairs catalytic function while only partially affecting secretion, in contrast to variants that markedly reduce both protein abundance and activity (Chan et al., 2000; Pingitore et al., 2016; Han et al., 2020; Guo et al., 2022).

Structurally, both variants are located within the C-terminal domain of LPL (**Figure 2**). For p.Glu396Val, replacement of a negatively charged glutamate by a hydrophobic valine may alter the local C-terminal structural environment or substrate-related enzymatic competence. However, because the ApoC-II cofactor-binding region is located primarily in the N-terminal domain of LPL, a direct effect on ApoC-II binding is unlikely (Kumari et al., 2023). Consistently, our LPL-GPIHBP1 interface-context analysi did not place Glu396 within 5 Å or 8 Å of GPIHBP1 in the resolved 6E7K structure (**Supplementary Figure 1B**), suggesting that the current structural evidence does not support direct disruption of the resolved GPIHBP1-binding interface.

For p.Trp417Cys, Trp417 lies within the WSDWW motif of the Trp-rich lipid-binding region encoded by exon 8 (**Figure 2**). This tryptophan-rich loop is crucial for lipid binding and lipoprotein interaction; therefore, replacement of a bulky aromatic tryptophan by cysteine may impair LPL interaction with lipid substrates or lipoprotein particles, providing an explanation for the reduced lipolytic activity (Lookene et al., 1997; Keiper et al., 2001). In addition, NetCGlyc predicted Trp417 as a potential C-mannosylation-related residue, and p.Trp417Cys removes this tryptophan site (**Supplementary Figure 1A**), suggesting a possible effect on residue-specific post-translational maturation (Okamoto et al., 2017). However, direct lipid-binding assays and biochemical assessment of LPL C-mannosylation were not performed; therefore, these mechanisms remain hypothesis-generating.

Overall, these findings support a model in which selected C-terminal missense variants reduce LPL enzymatic activity without markedly reducing protein abundance. The structural and bioinformatic analyses provide contextual support but do not directly establish altered conformational stability, defective dimerization, impaired GPIHBP1 binding, or disrupted C-mannosylation. The most direct evidence for variant impact remains the *in vitro* functional assay showing reduced LPL activity despite preserved mRNA and protein expression.

The marked reduction in triglyceride levels following improved metabolic control in proband 2 highlights the clinical principle that secondary metabolic stressors substantially modulate phenotypic expression in heterozygous *LPL* variant carriers. Insulin-based metabolic optimization may improve TG clearance by enhancing LPL activity and reducing VLDL production, and should be considered alongside conventional lipid-lowering therapy in patients with partial LPL dysfunction (Coskun et al., 2015; Thuzar et al., 2014). For individuals with refractory severe hypertriglyceridemia or recurrent pancreatitis, emerging targeted approaches (e.g., APOC3- or ANGPTL3-directed therapies) (Chen et al., 2024) may be considered in specialized settings, particularly when genetic testing identifies functionally significant variants.

Despite similar biochemical defects, the clinical presentation differed between subjects; notably, Proband 1 suffered recurrent pancreatitis while Proband 2 did not. The exceptionally high triglyceride level in Proband 2 is best interpreted as severe multifactorial hypertriglyceridemia rather than the consequence of a simple heterozygous *LPL* variant alone. Although p.Trp417Cys showed reduced LPL activity *in vitro*, its clinical expression was likely amplified by marked insulin resistance, diabetes, and hepatic steatosis. An additional oligogenic or polygenic background cannot be excluded because comprehensive polygenic risk assessment was not performed. Thus, genetic testing in severe HTG should be viewed not only as a tool for diagnosing rare monogenic chylomicronemia, but also as a means of identifying susceptibility variants that interact with metabolic stressors. This discrepancy remains a common challenge in LPL deficiency and likely reflects the influence of modifier genes or environmental factors not captured in this study.

Our study has limitations. First, due to patient safety concerns regarding heparin administration, post-heparin LPL activity was not measured *in vivo*. Second, our functional assays were performed using an overexpression system, which may not fully recapitulate physiological expression levels. Third, The LPL p.Glu396Val variant was also detected in Proband 1’s mother and sister, both with dyslipidemia, providing limited supportive segregation evidence. Familial testing was unavailable for Proband 2; therefore, the inheritance pattern of p.Trp417Cys could not be assessed. Fourth, Empty-vector and untransfected-cell controls were not included in the initial LPL activity assay; therefore, a minor contribution from endogenous lipase activity in the cell system or culture medium cannot be fully excluded.

## 5 Conclusion

In summary, our findings provide functional evidence that p.Glu396Val and the novel p.Trp417Cys impair LPL enzymatic activity despite preserved protein abundance, supporting a qualitative functional defect. These results expand the functional spectrum of C-terminal *LPL* variants and highlight the value of functional validation and metabolic assessment in interpreting heterozygous *LPL* variants in HTG.

## Data Availability

The datasets presented in this study can be found in online repositories. The names of the repository/repositories and accession number(s) can be found below: (Members, Cncb-Ngdc and Partners, 2025; Zhang et al., 2025): https://ngdc.cncb.ac.cn/gsa-human, HRA016152.
