# Supplementary material for: Reduced activity of two LPL C-terminal variants, p.Glu396Val and novel p.Trp417Cys: a clinical, biochemical and structural study
Source: Front Physiol. 2026 Jun 10;17:1820368. doi: 10.3389/fphys.2026.1820368 (PMC13290508; doi:10.3389/fphys.2026.1820368)
Supplement: Supplementary file 1 [file DataSheet1.docx]

**Supplementary**

**Table S1. Representative LPL C-terminal variants and associated clinical or functional consequences**

| **Variant** | **Nucleotide change** | **Exon** | **Protein region** | **Interpretation** | **Reported phenotype** | **Functional consequence / tolerance evidence** | **Reference / PMID** |
| --- | --- | --- | --- | --- | --- | --- | --- |
| p.Tyr329Ser | c.986A>C | Exon 6 | C-terminal domain, proximal C-terminal region | Likely pathogenic | Heterozygous proband with severe hypertriglyceridemia; familial segregation reported in affected relatives. | In vitro study in LO2 and TPC-1 cells: protein synthesis was not affected, but LPL activity was significantly reduced. | (Feng et al. 2022) |
| p.Arg333His | c.998G>A | Exon 6 | C-terminal domain | Pathogenic | Reported in familial chylomicronemia syndrome (FCS) / severe hypertriglyceridemia contexts, including compound heterozygosity with p.Gly215Glu and heterozygous severe HTG. | COS-cell functional study: near-complete loss of post-heparin LPL activity in cell medium and lysate. | (Rabacchi et al. 2015) |
| p.Met404Arg | c.1211T>G | Exon 8 | C-terminal domain; exon 8 region | Likely pathogenic | Homozygous FCS / type I hyperlipoproteinemia with very severe hypertriglyceridemia. | HEK293 functional study: near-abolished protein synthesis, ~80% reduction in secretion, and completely abolished catalytic activity; patient plasma showed extremely low LPL mass/activity. | (Pingitore et al. 2016) |
| p.Cys445Tyr | c.1334G>A | Exon 9 | Distal C-terminal domain; GPIHBP1 interaction-related region | Pathogenic | Reported in chylomicronemia / LPL-deficiency context. | Functional evidence showed impaired LPL binding to GPIHBP1, supporting a transport/interface-related mechanism for selected distal C-terminal variants. | (Voss et al. 2011) |
| p.Glu396Val | c.1187A>T | Exon 8 | C-terminal domain; exon 8 region | Current study variant; functionally deleterious | Proband 1: severe hypertriglyceridemia with recurrent acute pancreatitis and diabetes. | Current HEK293T assay: preserved LPL mRNA/protein abundance but significantly reduced enzymatic activity. | Current study |
| p.Trp417Cys | c.1251G>C | Exon 8 | Trp-rich / lipid-binding region, approximately residues 412–422 | Current study variant; novel and functionally deleterious | Proband 2: extreme hypertriglyceridemia, diabetes, severe insulin resistance, and fatty liver; no pancreatitis. | Current HEK293T assay: preserved LPL mRNA/protein abundance but significantly reduced enzymatic activity; may remove a potential C-mannosylation-related Trp residue by prediction. | Current study |
| p.Thr379Ile | c.1136C>T | Exon 7 | C-terminal domain, near exon 8 | Benign | Identified in a heterozygous severe hypertriglyceridemia patient; detailed clinical data were limited in the original report. | Functional data unavailable; classified as benign/tolerated because population frequency is higher than expected for monogenic LPL deficiency, including homozygous observations in population databases. | - (Rodrigues et al. 2016) |
| p.Ala427Thr | c.1279G>A | Exon 8 | C-terminal domain, distal to Trp-rich segment | Benign | Reported in FCS/severe HTG cohorts, but carriers with available data had normal post-heparin plasma LPL activity. | Normal post-heparin LPL activity in carriers and population frequency argue against monogenic pathogenicity. | (Surendran et al. 2012)  (Karczewski et al. 2020) |
| p.Ser474Term | c.1421C>G | Exon 9 | Distal C-terminal truncation | Benign / gain-of-function | Common variant associated in many studies with lower triglycerides or a favorable lipid profile. | Biochemical/functional evidence supports a gain-of-function or protective lipid effect; increased lipoprotein uptake has been reported. | (Hayne et al. 2017)  (Rip et al. 2006)  (Ranganathan et al. 2012)   - (Caussy et al. 2016) |

**
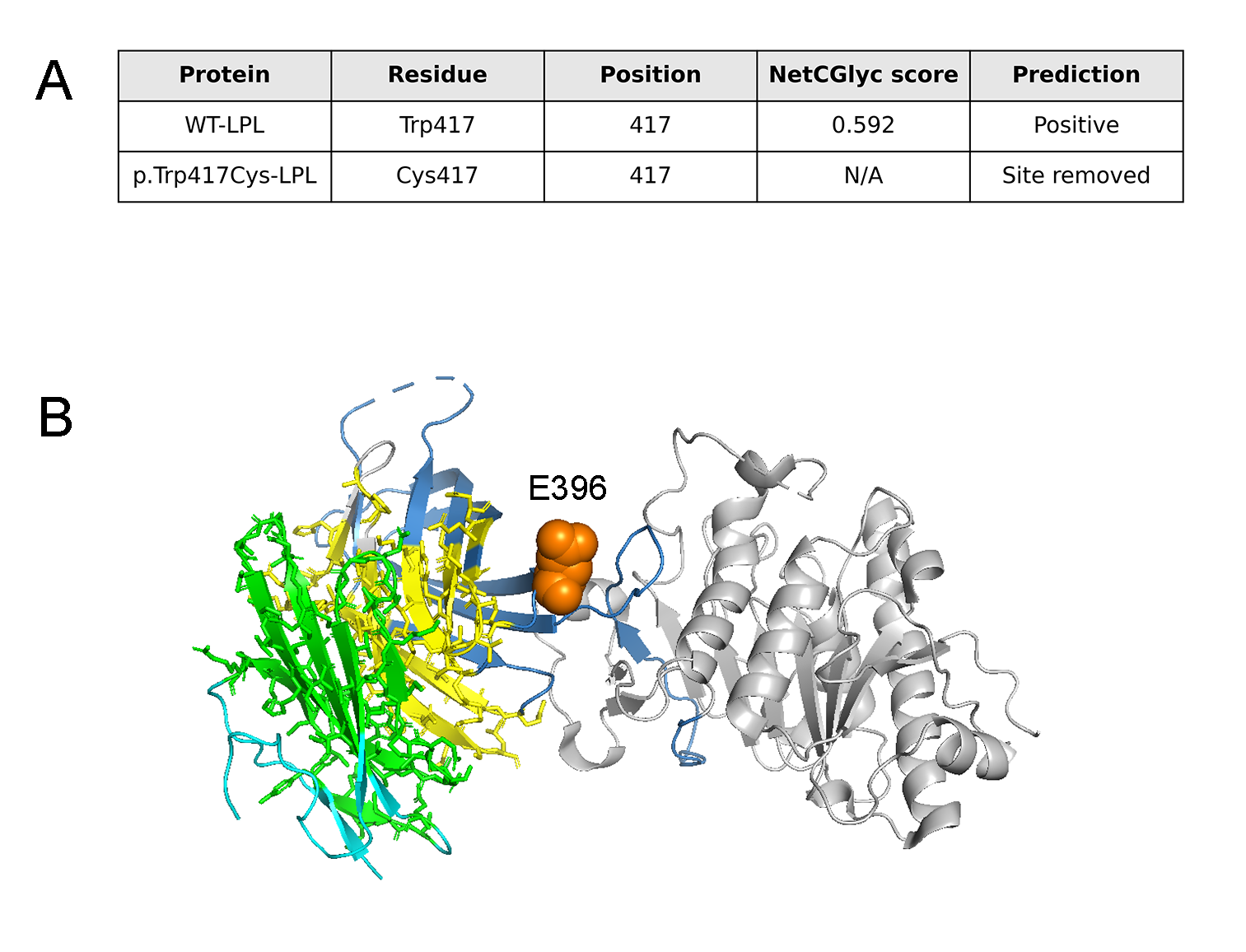
**

**Figure S1. NetCGlyc prediction and LPL–GPIHBP1 structural-context mapping.**

1. NetCGlyc prediction of potential C-mannosylation-related residues in LPL. NetCGlyc predicted Trp417 as a potential C-mannosylation-related tryptophan residue with a score of 0.592. According to the NetCGlyc output definition, scores range from 0.0 to 1.0, and residues with scores >0.5 are marked as positive predictions. The score represents algorithm-derived prediction confidence rather than a direct percentage probability of modification.
2. Mapping of Glu396 onto the experimentally determined LPL–GPIHBP1 complex structure. The LPL-GPIHBP1 complex structure was retrieved from the Protein Data Bank (PDB ID: 6E7K). PyMOL-based interface-proximity analysis showed that Glu396 was not located within 5 Å or 8 Å of GPIHBP1 in this static structure.

**References**

Caussy, C., S. Charriere, A. Meirhaeghe, J. Dallongeville, E. Lefai, S. Rome, C. Cuerq, V. Euthine, M. Delay, O. Marmontel, M. Di Filippo, M. Lagarde, P. Moulin, and C. Marcais. 2016. "Multiple microRNA regulation of lipoprotein lipase gene abolished by 3'UTR polymorphisms in a triglyceride-lowering haplotype harboring p.Ser474Ter." *Atherosclerosis* 246: 280-6. <https://doi.org/10.1016/j.atherosclerosis.2016.01.010>. <https://www.ncbi.nlm.nih.gov/pubmed/26820803>.

Feng, L., Y. Sun, F. Liu, C. Wang, C. Zhang, J. Liu, and L. Jiang. 2022. "Clinical features and functions of a novel Lpl mutation C.986A>C (p.Y329S) in patient with hypertriglyceridemia." *Curr Res Transl Med* 70 (4): 103337. <https://doi.org/10.1016/j.retram.2022.103337>. <https://www.ncbi.nlm.nih.gov/pubmed/35679768>.

Hayne, C. K., M. J. Lafferty, B. J. Eglinger, J. P. Kane, and S. B. Neher. 2017. "Biochemical Analysis of the Lipoprotein Lipase Truncation Variant, LPL(S447X), Reveals Increased Lipoprotein Uptake." *Biochemistry* 56 (3): 525-533. <https://doi.org/10.1021/acs.biochem.6b00945>. <https://www.ncbi.nlm.nih.gov/pubmed/27984852>.

Karczewski, K. J., L. C. Francioli, G. Tiao, B. B. Cummings, J. Alfoldi, Q. Wang, R. L. Collins, K. M. Laricchia, A. Ganna, D. P. Birnbaum, L. D. Gauthier, H. Brand, M. Solomonson, N. A. Watts, D. Rhodes, M. Singer-Berk, E. M. England, E. G. Seaby, J. A. Kosmicki, R. K. Walters, K. Tashman, Y. Farjoun, E. Banks, T. Poterba, A. Wang, C. Seed, N. Whiffin, J. X. Chong, K. E. Samocha, E. Pierce-Hoffman, Z. Zappala, A. H. O'Donnell-Luria, E. V. Minikel, B. Weisburd, M. Lek, J. S. Ware, C. Vittal, I. M. Armean, L. Bergelson, K. Cibulskis, K. M. Connolly, M. Covarrubias, S. Donnelly, S. Ferriera, S. Gabriel, J. Gentry, N. Gupta, T. Jeandet, D. Kaplan, C. Llanwarne, R. Munshi, S. Novod, N. Petrillo, D. Roazen, V. Ruano-Rubio, A. Saltzman, M. Schleicher, J. Soto, K. Tibbetts, C. Tolonen, G. Wade, M. E. Talkowski, Consortium Genome Aggregation Database, B. M. Neale, M. J. Daly, and D. G. MacArthur. 2020. "The mutational constraint spectrum quantified from variation in 141,456 humans." *Nature* 581 (7809): 434-443. <https://doi.org/10.1038/s41586-020-2308-7>. <https://www.ncbi.nlm.nih.gov/pubmed/32461654>.

Pingitore, P., S. M. Lepore, C. Pirazzi, R. M. Mancina, B. M. Motta, L. Valenti, K. E. Berge, K. Retterstol, T. P. Leren, O. Wiklund, and S. Romeo. 2016. "Identification and characterization of two novel mutations in the LPL gene causing type I hyperlipoproteinemia." *J Clin Lipidol* 10 (4): 816-823. <https://doi.org/10.1016/j.jacl.2016.02.015>. <https://www.ncbi.nlm.nih.gov/pubmed/27578112>.

Rabacchi, C., L. Pisciotta, A. B. Cefalu, D. Noto, R. Fresa, P. Tarugi, M. Averna, S. Bertolini, and S. Calandra. 2015. "Spectrum of mutations of the LPL gene identified in Italy in patients with severe hypertriglyceridemia." *Atherosclerosis* 241 (1): 79-86. <https://doi.org/10.1016/j.atherosclerosis.2015.04.815>. <https://www.ncbi.nlm.nih.gov/pubmed/25966443>.

Ranganathan, G., R. Unal, I. D. Pokrovskaya, P. Tripathi, J. I. Rotter, M. O. Goodarzi, and P. A. Kern. 2012. "The lipoprotein lipase (LPL) S447X gain of function variant involves increased mRNA translation." *Atherosclerosis* 221 (1): 143-7. <https://doi.org/10.1016/j.atherosclerosis.2011.12.028>. <https://www.ncbi.nlm.nih.gov/pubmed/22244040>.

Rip, J., M. C. Nierman, C. J. Ross, J. W. Jukema, M. R. Hayden, J. J. Kastelein, E. S. Stroes, and J. A. Kuivenhoven. 2006. "Lipoprotein lipase S447X: a naturally occurring gain-of-function mutation." *Arterioscler Thromb Vasc Biol* 26 (6): 1236-45. <https://doi.org/10.1161/01.ATV.0000219283.10832.43>. <https://www.ncbi.nlm.nih.gov/pubmed/16574898>.

Rodrigues, R., M. Artieda, D. Tejedor, A. Martinez, P. Konstantinova, H. Petry, C. Meyer, D. Corzo, C. Sundgreen, H. U. Klor, I. Gouni-Berthold, S. Westphal, E. Steinhagen-Thiessen, U. Julius, K. Winkler, E. Stroes, A. Vogt, P. Hardt, H. Prophet, B. Otte, B. G. Nordestgaard, S. S. Deeb, and J. D. Brunzell. 2016. "Pathogenic classification of LPL gene variants reported to be associated with LPL deficiency." *J Clin Lipidol* 10 (2): 394-409. <https://doi.org/10.1016/j.jacl.2015.12.015>. <https://www.ncbi.nlm.nih.gov/pubmed/27055971>.

Surendran, R. P., M. E. Visser, S. Heemelaar, J. Wang, J. Peter, J. C. Defesche, J. A. Kuivenhoven, M. Hosseini, M. Peterfy, J. J. Kastelein, C. T. Johansen, R. A. Hegele, E. S. Stroes, and G. M. Dallinga-Thie. 2012. "Mutations in LPL, APOC2, APOA5, GPIHBP1 and LMF1 in patients with severe hypertriglyceridaemia." *J Intern Med* 272 (2): 185-96. <https://doi.org/10.1111/j.1365-2796.2012.02516.x>. <https://www.ncbi.nlm.nih.gov/pubmed/22239554>.

Voss, C. V., B. S. Davies, S. Tat, P. Gin, L. G. Fong, C. Pelletier, C. D. Mottler, A. Bensadoun, A. P. Beigneux, and S. G. Young. 2011. "Mutations in lipoprotein lipase that block binding to the endothelial cell transporter GPIHBP1." *Proc Natl Acad Sci U S A* 108 (19): 7980-4. <https://doi.org/10.1073/pnas.1100992108>. <https://www.ncbi.nlm.nih.gov/pubmed/21518912>.
